# Building worlds of your own: the functional role of metacognitive bias in metacognition

**DOI:** 10.3389/fpsyg.2026.1792782

**Published:** 2026-07-01

**Authors:** Domenico Romanazzi, Anna Pasini, Luca Tarasi, Vincenzo Romei

**Affiliations:** 1 Centro Studi e Ricerche in Neuroscienze Cognitive, Dipartimento di Psicologia “Renzo Canestrari”, Alma Mater Studiorum – Università di Bologna, Campus di Cesena, Cesena, Italy; 2 Dipartimento di Filosofia, Alma Mater Studiorum – Università di Bologna, Bologna, Italy; 3 Universidad Antonio de Nebrija, Madrid, Spain

**Keywords:** confidence, confirmation bias, metacognitive bias, metacognitive sensitivity, meta-criterion, confidence bias measure

## Abstract

Metacognition, the capacity to monitor and evaluate one’s own decisions, has become a central topic in psychological and neuroscientific research. While research has largely focused on metacognitive sensitivity, defined as the ability to discriminate between correct and incorrect decisions, considerably less attention has been devoted to metacognitive bias, defined as the systematic tendency to report higher or lower confidence irrespective of objective accuracy. Here, we argue that this imbalance has limited current theories of metacognition. Instead, greater focus on metacognitive bias can deepen our understanding of internal models of confidence, revealing how inter-individual differences give rise to distinct internal representations of the world, even when external information is processed in comparable ways. This perspective reframes metacognitive bias as a regulatory mechanism that balances flexibility and stability in self-evaluation, rather than a failure of calibration. Given its theoretical and empirical relevance, we emphasize the importance of estimating this construct within a Signal Detection Theory (SDT) framework. We propose the meta-criterion (meta-c), derived from the model introduced by Maniscalco and Lau, as a principled and quantitative index of metacognitive bias. Unlike purely descriptive measures, meta-c captures fine-grained individual differences along a continuous scale, opening to the possibility of defining more conservative versus more liberal confidence policies. We foresee metacognitive bias as one of the central axes along which the next generation of metacognitive research will develop.

## 1 Introduction

It is pouring rain late in the evening as you drive toward a party. On the way, you are supposed to pick up a friend who told you exactly where he’d be waiting, by the side of the road. As you near the spot, you think you can make out a figure through the blur of rain and darkness. The place matches the description, so you slow down and roll down the window. But how sure are you that the person standing there is really your friend? You know he’s supposed to be there, yet the weather makes it almost impossible to tell who’s actually standing in the downpour.

Making a perceptual decision under uncertain conditions can be challenging and may sometimes lead to error. Fortunately, we can become aware of such uncertainty, as the perceptual decision-making process does not end with the initial choice but continues into a metacognitive stage, during which we evaluate and reflect upon the accuracy of our preceding decision (Fleming, 2017). The process by which individuals evaluate the accuracy of a decision they have just made is referred to as metacognition, defined as the capacity to understand and monitor one’s own cognitive processes (Flavell, 1979). In everyday life, we continuously engage in such processes, sometimes consciously and sometimes automatically, giving rise to a level of confidence in our decisions.

Confidence levels should faithfully reflect the accuracy of our decisions: ideally, individuals would report high confidence following correct choices and low confidence after incorrect ones. In practice, however, maintaining such calibration requires considerable cognitive resources. Metacognitive monitoring operates continuously across decision processes, rather than being engaged only when explicit confidence judgments are required, thereby imposing a considerable computational load on the limited-capacity human brain. As a result, confidence judgments are not perfectly objective but are instead biased by internal noise, cognitive biases, clinical conditions and aging (Seow et al., 2025; Rouault et al., 2018; McWilliams et al., 2023).

From a theoretical standpoint, within the framework of Signal Detection Theory (SDT; Green and Swets, 1966), in perceptual judgments, commonly referred to as Type I decisions, one can distinguish between sensitivity (d’), which measures an individual’s ability to discriminate relevant sensory information from background noise, and the decision criterion (c), which reflects a systematic bias or predisposition toward a particular response. An analogous distinction applies to metacognitive judgments, or Type II decisions (Clarke et al., 1959; Galvin et al., 2003). Specifically, metacognitive sensitivity (meta-d’) quantifies an individual’s ability to discriminate between correct and incorrect perceptual decisions, while the metacognitive criterion (meta-c) quantifies a general metacognitive bias representing the threshold along the internal evidence axis for reporting higher or lower confidence independent of metacognitive sensitivity (Maniscalco and Lau, 2012; Maniscalco and Lau, 2014; Fleming and Lau, 2014; See Section 3 for details).

Recent work in metacognitive psychology and neuroscience has increasingly focused on metacognitive sensitivity and can be broadly framed as comprising two main research trajectories (Fleming, 2024). One line of research examines how metacognitive sensitivity could be dissociated from Type I accuracy (Zylberberg et al., 2012; Zylberberg et al., 2014; De Gardelle and Mamassian, 2015; Spence et al., 2016; Constant et al., 2023; Di Gregorio et al., 2022; Di Luzio et al., 2022; Maniscalco and Lau, 2016; Rahnev et al., 2012; Tarasi et al., 2025; Di Luzio et al., 2025; Trajkovic et al., 2024); while, another line of research aims to delineate the neural processes that support accurate confidence judgments (Fleming et al., 2010; Baird et al., 2013; De Martino et al., 2013; Desender et al., 2016; Kiani and Shadlen, 2009; Hebart et al., 2016; Yeon et al., 2020).

Together, these lines of research have substantially advanced our understanding of metacognitive sensitivity and its determinants, while metacognitive bias has received comparatively limited attention, a gap reflected also in the methods used to assess it (Tarasi et al., 2025; Xue et al., 2021; Zajkowski et al., 2022; Culot et al., 2023; Olawole-Scott and Yon, 2023). Indeed, numerous computational models have been developed and systematically compared to explain metacognitive sensitivity (Yeon et al., 2020); by contrast, metacognitive bias has rarely been examined within a model-based framework. In this regard, whereas metacognitive sensitivity is typically assessed using the SDT model, metacognitive bias has most often been operationalized as mean confidence (Fleming and Lau, 2014), defined as the average confidence rating across both correct and incorrect responses. Although this measure is easy to compute, its very simplicity may have contributed to the relative neglect of adequate computational models to estimate metacognitive bias. In the following sections, we argue that the theoretical and empirical relevance of metacognitive bias demands a more rigorous modeling approach. To anticipate, we propose the SDT model (Maniscalco and Lau, 2012; Maniscalco and Lau, 2014), already widely used to estimate metacognitive sensitivity, should also serve as the gold standard for measuring metacognitive bias.

## 2 The importance of bias

Every time we make a choice, the process of evaluating that decision plays a crucial role. This evaluation allows for a “re-examination” of the choice that has been made and, consequently, for the adjustment of future behavior. In this sense, individuals continuously interpret the external world, and the quality of this interpretation determines how their read-out internal model of the world (i.e., an internal representation integrating prior knowledge and expectations about incoming events) is updated. Such interpretation can be parameterized through metacognitive sensitivity, understood as the ability to accurately discriminate between correct and incorrect responses based on subjective confidence.

However, is updating the internal model always possible, and, more importantly, always beneficial? The first fundamental property of an adaptive model is flexibility: an overly rigid model cannot be effectively updated. At the same time, when the internal model reflects the complexity of human behavior in an inherently chaotic environment, continuous and unselective updating may lead to persistent instability, thereby undermining a fundamental state for human beings: internal coherence (Tarasi et al., 2022).

From this perspective, metacognitive bias may serve a self-consistency function by regulating updates to the internal model (Caziot and Mamassian, 2021). By “self-consistency,” we refer to the tendency of the cognitive system to maintain a stable relationship between internal beliefs and external evidence, even when moment-to-moment fluctuations in performance would warrant recalibration. This does not imply that the system ignores evidence, but rather that it filters and integrates evidence through a relatively stable confidence policy (i.e., the metacognitive criterion, namely the point along the decisional continuum at which an individual sets their bias when expressing confidence judgments), thereby reducing the computational cost of continuous re-evaluation.

The phenomenon of confirmation bias provides a clear illustration of this balance. It refers to the systematic tendency to prioritize information that supports preexisting beliefs while downplaying or disregarding evidence that contradicts them (Nickerson, 1998). This heuristic can be interpreted in both negative and positive terms. In a world that constantly demands decisions, often made rapidly and under high cognitive load, the cognitive “shortcut” offered by confirmation bias may constitute a functional adaptation. Rather than continuously recalibrating confidence to match fluctuating accuracy, individuals may adopt a stable confidence policy that minimizes the computational burden of metacognitive evaluation. Moreover, imposing one’s own model may favor its own implementation over alternative ones, certainly providing adaptive advantage (Sharot, 2011). Consistent with this view, metacognitive bias may be particularly functional in preserving self-consistency when decisions must be evaluated against strong prior expectations: under such conditions, it was demonstrated that confidence can be systematically modulated by the congruence between prior expectations and perceptual decisions, reinforcing internal consistency, while metacognitive sensitivity remains unaffected (Tarasi et al., 2026).

From this perspective, metacognitive bias does not merely reflect a deviation from an objective standard, but rather a stable characteristic of how individuals regulate the relationship between internal beliefs and external evidence. Consistent with recent theoretical accounts, this regulation may operate through the differential weighting of internal cues, such as the coherence among cues sampled at the moment of decision (Koriat, 2024) and heuristic signals such as fluency or prior beliefs (Schwartz, 2024), relative to external evidence. Metacognitive bias, in this sense, functions as a relatively stable policy that determines how readily incoming evidence is allowed to update confidence. Such biases may therefore serve an adaptive function, promoting self-consistency and preserving homeostatic balance in environments characterized by uncertainty and informational overload (Sharot, 2011). In line with this view, high confidence in a decision has been shown to directly reduce subsequent belief updating (Rollwage et al., 2020), suggesting that, by limiting excessive model updating, biased confidence readouts may protect the internal model from instability, allowing behavior to remain coherent over time (Rollwage and Fleming, 2021).

When examined within the psychopathological domain, metacognitive biases have been shown to systematically manifest and contribute to a range of conditions, including anxiety, depression, and obsessive-compulsive disorder (OCD; Seow et al., 2025; Rouault et al., 2018). In particular, anxious and depressive symptomatology has been consistently associated with a pattern of underconfidence, whereas OCD has been linked to overconfidence, both in perceptual and memory-related tasks. Recent evidence further clarifies the mechanisms through which these biases may persist over time: a globally underconfident metacognitive state (e.g., global self-performance estimates) has been shown to reduce responsiveness to instances of relatively high local confidence (e.g., moment to moment confidence), thereby constraining the updating of self-evaluative performance estimates (Katyal et al., 2025). Furthermore, within anxiety, prolonged introspective processing has been associated with the gradual accumulation of a negative metacognitive bias, which in turn amplifies underconfidence over time (Katyal and Fleming, 2026). Moreover, an underconfidence bias has also been observed in older adults compared to younger populations (McWilliams et al., 2023), suggesting that alterations in metacognitive bias may reflect broader changes in confidence calibration across both clinical and non-clinical older populations.

Importantly, within the self-consistency framework, these patterns may reflect a rigid and inadequate manifestation of the mechanism that balances prior beliefs and sensory evidence. Inadequate integration of external evidence can give rise to distorted metacognitive states, that in a self-reinforcing vicious cycle, can sustain psychopathological symptomatology (as well as the cognitive changes associated with aging), exposing individuals to the risks associated with perceiving themselves as either less or more confident than warranted by both prior knowledge and external evidence.

The unifying concept underlying these metacognitive “distortions” is that variations in the internal confidence model can occur independently of changes in metacognitive sensitivity.

Some individuals consistently exhibit high confidence in their decisions, whereas others systematically report lower confidence, thus positioning themselves along a continuous dimension of internal confidence models. Crucially, although distinct internal models may ultimately yield comparable levels of metacognitive sensitivity, adopting a systematically “optimistic” or “pessimistic” interpretive stance can shape the perceived quality of one’s actions, judgments, and experiences (Sharot, 2011).

**Figure 1:**
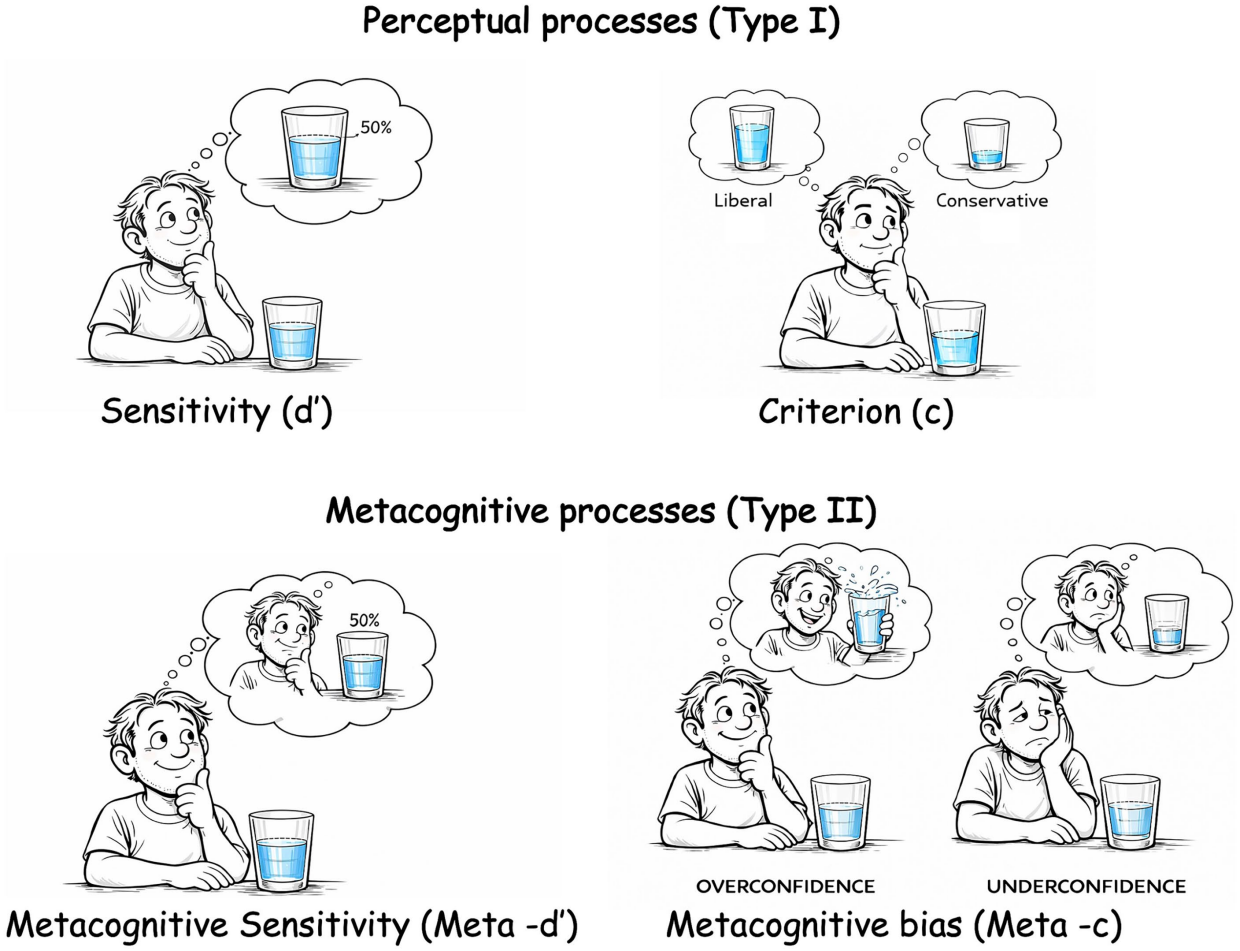
The figure illustrates a hierarchical Signal Detection Theory (SDT) framework distinguishing first-order (Type I) perceptual processes from second-order (Type II) metacognitive processes. In perceptual processes (Type I), the sensitivity (d′) reflects the quality of the internal evidence extracted from the stimulus (here, a glass containing a fixed, objective amount of water) and used to form the first-order decision. Based on this internal evidence, the criterion (c) determines how perceptual decisions are formed, such that identical sensory input can lead to different categorical perceptual reports depending on the placement of the decision threshold (e.g., more liberal versus more conservative criteria). For example, a more liberal criterion may lead one to perceive a glass as half full, whereas a more conservative criterion may lead one to perceive the same glass, containing the same amount of water, as half empty. Importantly, variability at the Type I level reflects differences in decision thresholds rather than distortions of sensory evidence. The output of Type I processing feeds into Type II metacognitive monitoring, where metacognitive sensitivity (meta-d′) captures how well confidence judgments discriminate between correct and incorrect first-order decisions. Crucially, beyond metacognitive sensitivity, the metacognitive criterion (meta-c) plays a central role in shaping subjective confidence. Systematic shifts in this criterion determine whether individuals tend toward overconfidence (optimistic bias: believing the glass to be fuller than it actually is) or underconfidence (pessimistic bias: believing it to be emptier than it actually is), independently of both sensitivity (d′) and metacognitive sensitivity (meta-d′). As a result, this metacognitive bias, by exerting control over the filtering and integration of sensory evidence, can lead to stable and systematic divergences between objective performance and subjective confidence, highlighting its critical importance in understanding individual differences in self-evaluation, decision awareness, and adaptive behavior.

In this light, increasing emphasis on the study of metacognitive bias, beyond traditional measures of metacognitive sensitivity, offers a crucial opportunity to better understand how different clinical and non-clinical populations can maintain comparable levels of objective performance while nonetheless constructing systematically distinct representations of the world (Figure 1).

## 3 Metacognitive model

In experimental settings, one of the most widely adopted methods to elicit confidence is through retrospective judgments, whereby participants are asked to indicate, on a Likert scale (e.g., 0 = not confident at all, 4 = highly confident), how certain they are about the accuracy of their Type I decision (Fleming and Dolan, 2012). Ideally, a subject with good metacognitive ability expresses high confidence when he is correct and low confidence when he is not. Once confidence ratings are obtained, different approaches can be employed to quantify individual levels of metacognition.

One method evaluates the extent to which confidence judgments align with actual accuracy, calculating the proportion of correct and incorrect responses followed by high or low confidence judgments (Persaud et al., 2007). Another method involves computing the association between confidence ratings and accuracy, typically using either the Pearson correlation coefficient (*φ*) or the Goodman–Kruskal gamma coefficient (Goodman and Kruskal, 1954; Xue et al., 2021; Zajkowski et al., 2022; Nelson, 1984).

Nevertheless, this method is fundamentally constrained by its inability to effectively dissociate metacognitive sensitivity from metacognitive bias, thus rendering the two metrics potentially confounded (Maniscalco and Lau, 2012). This occurs because these measures typically rely on raw confidence ratings, which simultaneously reflect how well participants can discriminate between correct and incorrect decisions (metacognitive sensitivity) and their general tendency to report higher or lower confidence irrespective of performance (metacognitive bias). As a result, changes in confidence ratings can arise either from genuine improvements in the ability to track one’s own accuracy or simply from shifts in the overall level of confidence, making it impossible to determine which component is driving the observed effects.

In light of these limitations, methodologies grounded in Signal Detection Theory (Culot et al., 2023) are regarded as the preferred and more robust approach for the quantification of confidence, as they allow for a clear and explicit separation between the two parameters. Therefore, building on Galvin’s conceptualization (Galvin et al., 2003), Maniscalco and Lau (2012) introduced a Type II Signal Detection Theory (SDT) framework that models metacognitive sensitivity on the basis of Type I performance. Within this framework, it is possible to estimate how an ideal observer would use Type I information to generate Type II data. The resulting parameter (meta-d’), representing metacognitive sensitivity, can be interpreted as an index of how much signal is available to the observer when making Type II decisions.

The proposed model represents an advance in metacognitive research, as it allows metacognitive sensitivity and metacognitive bias to be evaluated independently. Although this framework is now widely employed to quantify metacognitive sensitivity, the same cannot be said for metacognitive bias. Indeed, many studies that have measured confidence and estimated metacognitive sensitivity using this framework have nonetheless quantified metacognitive bias using mean confidence, rather than parameters derived from the same model (Tarasi et al., 2025; Xue et al., 2021; Zajkowski et al., 2022; Culot et al., 2023; Olawole-Scott and Yon, 2023).

Although mean confidence is simple to compute, we suggest that adopting the same modeling framework used for meta-d’ (Maniscalco and Lau, 2014) would provide several interpretative advantages. Indeed, within this framework, it is also possible to estimate an additional parameter that allows the quantification of metacognitive bias: the meta-criterion (meta-c).

This metric parallels the Type I decision criterion but is derived by grouping trials according to confidence and accuracy rather than by stimulus class and behavioral response. The estimation of this parameter, using either a classical (Maniscalco and Lau, 2012; Maniscalco and Lau, 2014) or a Bayesian (Fleming, 2017) approach, involves identifying the set of thresholds along the internal evidence axis that best account for the observed distribution of confidence ratings, given the estimated meta-d’ (Fleming, 2017; Maniscalco and Lau, 2012; Maniscalco and Lau, 2014).

Due to its structure, the SDT model provides k-1 meta-criteria for each response (absent, present), where “k” represents the number of confidence levels a participant can express. For example, if the task allows a participant to report k = 4 confidence levels, the model yields 3 meta-criteria (per response). This makes the characterization of metacognitive bias less straightforward than that of metacognitive sensitivity (meta-d’), which is summarized by a single parameter. One possible solution is to collapse the confidence ratings from the outset into a binary choice, high versus low confidence (Maniscalco and Lau, 2014; Maniscalco et al., 2025), regardless of the original scale resolution (e.g., collapsing 1–2 vs. 3–4 in a 4-point scale, or 0–50 vs. 50–100 in a continuous scale) allowing for the estimation of a single pair of confidence criteria (one for each response: absent and present).

Subsequently, the two values calculated using the approach proposed by Sherman et al. (see below), can be summed or averaged to yield a single, response-independent index of metacognitive bias (Benwell et al., 2022), making it as intuitive and interpretable as meta-d′. It should be noted that this approach is not always a neutral operation, as it may involve some loss of information. Nonetheless, alternative approaches are also available and represent valid strategies for characterizing metacognitive bias (Benwell et al., 2022). Future research should further investigate methodological solutions capable of estimating metacognitive criteria while minimizing information loss.

The main advantage of using this model is the possibility to isolate metacognitive bias from Type I decisional bias (O’Neill et al., 2026; Sherman et al., 2018). In fact, the level of confidence reported by an individual is strongly influenced by the decision strategy adopted in the task, that is, by the position of the Type I criterion (c). A participant with a highly conservative criterion will respond “signal present” only when the internal evidence is particularly strong; consequently, those ‘present’ responses will almost always be accompanied by low confidence. In other words, a strong tendency to respond conservatively can, by itself, produce an apparent “confidence bias,” even in the absence of any additional metacognitive bias. By computing metacognitive bias as the absolute distance between each meta-criterion and the corresponding Type-I criterion, all normalized by the individual’s meta-d’ (Sherman et al., 2018), it becomes possible to quantify a metacognitive bias that does not depend on the response strategies adopted in the Type I task. This procedure yields two positive bias values (one per response category), each reflecting the magnitude of metacognitive bias displacement independently of the direction of the type-I decision. The normalization by individual’s meta-d’ yields a scale-invariant measure of criterion placement, allowing comparisons across subjects with different levels of metacognitive sensitivity. This may allow us to overcome some of the interpretational limits of descriptive confidence measures, providing a potentially more faithful representation of metacognitive bias as a property of the internal monitoring system that is less confounded by the decision strategies used in the task (Benwell et al., 2022; Sherman et al., 2018).

Another advantage of this approach is the possibility of estimating separate meta-criteria for different response types, for example, “signal present” and “signal absent” in a detection task. This refinement is motivated by the fact that the processes underlying confidence judgments may not be homogeneous across all trial types (Maniscalco and Lau, 2012; Maniscalco and Lau, 2014). By allowing a distinct meta-criterion for each case, the model provides a more nuanced characterization of metacognitive performance, capturing potential asymmetries in how confidence is generated and expressed. Importantly, this measure is also highly intuitive and can be readily interpreted without a detailed understanding of SDT.

In this context, meta-criteria closer to zero reflects a more liberal confidence criterion, meaning that participants are more inclined to report high confidence for the corresponding response. Conversely, more distant values indicate a conservative criterion, reflecting a greater reluctance to endorse high confidence (Fleming and Lau, 2014). Moreover, because this parameter can, in principle, assume values from negative to positive infinity, unlike mean confidence, which is constrained to a limited range (typically from 1 to 4; Fleming and Lau, 2014), it provides a fine-grained, continuous measure for characterizing individual metacognitive strategies and quantifying inter-individual differences along an internal readout model continuum. The growing recognition of the importance of metacognitive bias further underscores the need for a well-established and powerful model to serve as a direct measure of such distortions. Unlike mean confidence, which is purely descriptive, meta-c situates metacognitive bias within a rigorous signal detection theoretical framework, allowing for a more precise, model-based characterization of the underlying cognitive processes.

To be fair, it should be acknowledged that the field has moved considerably beyond meta-d′ since its introduction (Rahnev, 2021; Katyal and Fleming, 2024) and that this measure relies on assumptions that have increasingly come under scrutiny (Shekhar and Rahnev, 2021a; Shekhar and Rahnev, 2021b). Our intention here is not to advocate for Type-II SDT measures as definitive measures of metacognition, but rather to make a more focused argument: when meta-d′ is used, as it remains the most widely adopted measure in the literature, its typical companion index of bias, namely mean confidence, is suboptimal, whereas meta-c offers a more principled alternative within the same SDT framework.

Moreover, recent advances in the computational modeling of metacognitive bias, particularly through DDM-based approaches (Katyal and Fleming, 2026; Van Marcke et al., 2024), provide complementary perspectives to SDT-based accounts. While such models are especially well suited to characterizing the temporal dynamics and generative mechanisms underlying confidence formation, SDT-based approaches provide a more direct characterization of where within the metacognitive system the bias may reside.

## 4 Brief demonstration of differences between the usage of mean confidence and meta-c

To further evaluate the relative specificity of meta-c compared with simpler descriptive measures of confidence, we analyzed data from our dataset (Tarasi et al., 2026). The data were collected in a perceptual detection task in which participants reported the presence or absence of a target stimulus. Each trial began with a cue indicating the prior probability of target presence (low: 33%, random: 50%, high: 67%). Participants were explicitly informed that the actual target probability matched the cue. Following each perceptual decision, participants provided a confidence rating on a 1–4 scale.

Because prior probability was shown to modulate confidence ratings, and in order to obtain unbiased and computationally comparable estimates, we restricted the present analyses to trials following the random prior condition (50%), obtaining a final sample of 119 participants. Mean confidence and meta-c were computed separately for “present” and “absent” responses and then averaged across conditions to yield a single value per participant. Meta-c was averaged after transposing the values associated with “absent” responses, which are predominantly negative due to the SDT convention, onto the positive axis. This transformation allowed the computation of a unified mean meta-c, such that smaller values consistently reflected a more liberal average meta-c. In addition, we computed d’ and meta-d’. To directly test the advantage of meta-c, we computed Pearson correlations between sensitivity (d’, meta-d’, m-ratio), bias (Type I c) and confidence (mean confidence and meta-c) measures. We also examined the correlation between the two confidence measures. The two confidence metrics were strongly correlated with each other (*r* = −0.89 *p* < 0.05).

Mean confidence showed a significant positive correlation with meta-d′ (*r* = 0.37, *p* < 0.05), with d’ (*r* = 0.29, *p* < 0.05), and with m-ratio (*r* = 0.20, *p* < 0.05) whereas meta-c was not associated with either measure (all |r| < = 0.04, all p > = 0.65, BF_10_ < = 0.127). These findings indicate that mean confidence increases with perceptual sensitivity and, consequently, with metacognitive sensitivity indices. In contrast, meta-c may provide a measure which is preferable to mean confidence to quantify metacognitive bias, as it reduces the influence of differences in discrimination performance. Consistent evidence for such an association between mean confidence and metacognitive efficiency has also been reported by Xue et al. (2021), who highlighted this relationship as a potential confound when the two measures are jointly interpreted across individuals and tasks.

**Figure 2:**
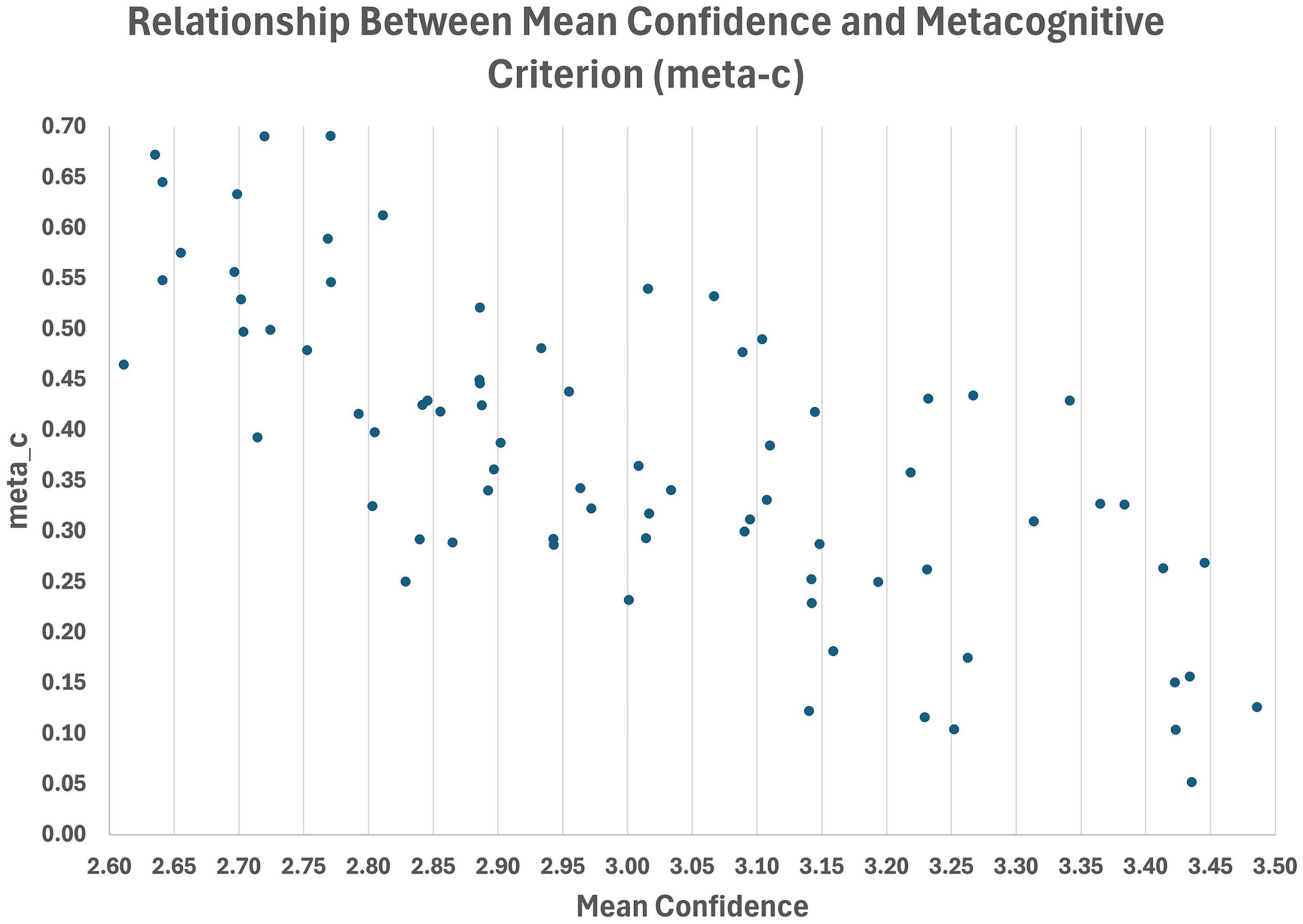
The figure shows the relationship between confidence mean and meta-c. It clearly emerges that, at equal levels of confidence mean, different values of meta-c can correspond, indicating that this measure can capture more fine-grained differences between subjects.

This pattern of results is, in fact, theoretically expected. As perceptual sensitivity increases, the proportion of correct responses increases, and the proportion of errors decreases. Higher sensitivity leads to a greater proportion of high-confidence correct responses and fewer low-confidence incorrect responses. This asymmetry systematically biases mean confidence upward, as the aggregation of predominantly high ratings inflates the average. By contrast, meta-c is estimated within a signal detection framework that accounts for hit rate and false alarm rate, possibly reducing contamination from sensitivity-related differences in response distributions. Furthermore, when examining the relationship with the Type I criterion separately for absent responses and present responses (Sherman et al., 2018), a positive relationship emerges between mean confidence for absent responses and c (*r* = 0.25, *p* < 0.05), along with a strong trend toward a negative association between mean confidence for present responses and c (*r* = −0.15, *p* = 0.10), thereby replicating Sherman et al.’s findings. Regarding meta-c, a clear relationship with the Type I criterion is also observed for both present and absent responses (all r > = 0.74, all *p* < 0.05), as already highlighted by Sherman et al. (2018). This confound can be readily addressed by correcting meta-c for the influence of the Type I criterion (Benwell et al., 2022; Sherman et al., 2018), thereby yielding a measure that is unbiased with respect to the Type I decision criterion (all |r| < = 0.07, all *p* > = 0.42). Finally, as shown in Figure 2, identical values of mean confidence can correspond to different values of meta-c. This represents an advantage of the meta-c measure over mean confidence, as it may allow for a more fine-grained characterization of individual differences that may not be captured by a coarser metric. This increased granularity may also translate into greater sensitivity in detecting effects that would otherwise remain undetected using simpler summary measures (Sherman et al., 2018). However, these findings may still be partly influenced by the specific experimental paradigm employed. Future work will therefore be needed to assess their generalizability across independent datasets and alternative task designs.

## 5 Discussion

Metacognition enables individuals to monitor their cognitive processes and to generate confidence judgments that guide behavior and learning (Khalvati and Rao, 2015; Mamassian, 2016). When confidence is well calibrated, it supports adaptive decision-making by facilitating appropriate behavioral adjustments. However, achieving perfect calibration is computationally demanding, and in everyday contexts, cognitive resources are often limited. Under such conditions, individuals cannot allocate full evaluative effort to every metacognitive judgment and therefore rely on heuristic processes that systematically bias confidence reports.

Crucially, these biases should not be interpreted as mere metacognitive failures. Rather, they may emerge as adaptive shortcuts that allow the cognitive system to conserve resources while maintaining internal coherence (Caziot and Mamassian, 2021; Sharot, 2011; Rollwage and Fleming, 2021). Instead of engaging in effortful re-evaluation of performance, individuals may rely on heuristic cues or prior beliefs when forming confidence judgments, thereby stabilizing their internal model without necessarily altering objective performance.

Nonetheless, the same shortcuts that promote efficiency may become maladaptive when they persist across contexts or disproportionately influence self-evaluation. Systematic overconfidence can lead to risky or impulsive decisions, whereas chronic underconfidence may hinder performance, generating hesitation and doubt even in the presence of correct judgments (Seow et al., 2025; Rouault et al., 2018). These opposing tendencies illustrate how metacognitive biases shape the subjective interpretation of reality, influencing not only what individuals believe about their performance but also how they experience and regulate their behavior.

Recognizing both the functional and dysfunctional aspects of metacognitive bias motivates a conceptual shift in metacognitive research. Metacognitive bias can be interpreted as a regulatory mechanism that stabilizes the read-out internal model of the world, balancing the need for flexibility and updating against the preservation of internal coherence and homeostasis. Furthermore, given its relevance, we suggest that traditional descriptive indices, such as mean confidence or confidence–accuracy correlations, offer limited insight into the underlying structure of confidence formation. By contrast, model-based approaches provide a principled framework for disentangling the components of metacognitive evaluation. Within a signal detection theoretical framework, the parameter meta-c offers a formal quantification of metacognitive bias, capturing systematic shifts in the placement of the internal confidence criterion. Importantly, meta-c situates these shifts along a continuous dimension, ranging from conservative to liberal confidence policies, rather than reducing them to aggregate descriptive statistics.

Embedding metacognitive bias within this theoretical structure further allows a critical distinction between metacognitive sensitivity (meta-d’) and subjective confidence policy (meta-c). This distinction reflects a deeper principle: individuals may exhibit comparable levels of metacognitive sensitivity while relying on qualitatively different internal read-out models of their cognitive performance. Some observers may adopt an optimistic, liberal confidence stance, whereas others may operate under a more conservative, self-monitoring regime. Examining metacognitive bias through parameters such as meta-c therefore provides direct access to the internal representation of the external world, revealing how different individuals construct and regulate it.

Taken together, this perspective reframes metacognitive bias not as a secondary artifact of poor performance, but as a core dimension of metacognitive architecture, intimately linked to the maintenance of self-stability. Systematic biases in confidence may contribute to preserving a coherent sense of self across time and contexts, shielding the individual from excessive fluctuations in self-evaluation. Understanding how confidence is systematically biased, therefore, has important implications for both basic and applied research, offering a unified framework for interpreting variability in self-consistency across normative, clinical, and aging populations, even in the presence of comparable levels of objective accuracy.

## Data Availability

The original contributions presented in the study are included in the article/supplementary material, further inquiries can be directed to the corresponding authors.
